# Meteorological Report for March

**Published:** 1880-04

**Authors:** 


### METEOROLOGICAL REPORT FOR MARCH, 1880.

| RAIN. DATE | IN. | Mean daily Barometer. | Mean daily Thermometer | Mean daily Humidity. | Maximum Thermometer | Minimum Thermometer | Prevailing direction of Wind. | Maximum hourly velocity of Wind, (Daily.) |
|---|---|---|---|---|---|---|---|---|
| 1 | 02 | 30.172 | 53.5 | 71.0 | 60 | 40 | N. W. | ......... |
| 2 | 0 | 30.306 | 54.7 | 52.3 | 64 | 46 | E. | ......... |
| 3 | 06 | 30.099 | 57.5 | 78.0 | 63 | 49 | S. E. | ......... |
| 4 | 0 | 29.891 | 69.0 | 70.0 | 75 | 62 | S. W. | ......... |
| 5 | 15 | 29.903 | 69.7 | 72.0 | 70 | 64 | S. W. | ......... |
| 6 | 0 | 29.904 | 68.5 | 79.3 | 73 | 59 | S. W. | ......... |
| 7 | 0 | 29.982 | 66.5 | 66.0 | 78 | 61 | S. W. | ......... |
| 8 | 1.12 | 29.969 | 58.5 | 87.6 | 63 | 53 | S. | ......... |
| 9 | 94 | 29.942 | 55.5 | 89.6 | 64 | 45 | N. E. | ......... |
| 10 | 01 | 29.997 | 55.7 | 75.3 | 62 | 47 | S. E. | ......... |
| 11 | 1.45 | 29.979 | 56.2 | 73.3 | 61 | 52 | E. | ......... |
| 12 | 1.30 | 30.027 | 52 2 | 93.0 | 64 | 42 | E. | ......... |
| 13 | 1.71 | 30.128 | 42.5 | 91.6 | 45 | 40 | N. E. | ......... |
| 14 | 79 | 30.116 | 51.5 | 60.3 | 57 | 40 | W. | ......... |
| 15 | 1.63 | 29.944 | 57.5 | 89.0 | 60 | 53 | E. | ......... |
| 16 | 1.37 | 29.934 | 53.2 | 83.3 | 62 | 46 | W. | ......... |
| 17 | 0 | 30.159 | 44.5 | 57.3 | 57 | 35 | N. W. | ......... |
| 18 | 1.00 | 29 965 | 49.2 | 74.0 | 60 | 42 | E. | ......... |
| 19 | 24 | 39.985 | 53.5 | 71.3 | 60 | 45 | N. W. | ......... |
| 20 | 0 | 30.193 | 53.2 | 41.3 | 60 | 44 | N. W. | ......... |
| 21 | 0 | 30.227 | 57.2 | 39.3 | 62 | 47 | N. W. | ......... |
| 22 | 0 | 30.123 | 57.8 | 43.7 | 65 | 50 | W. | ......... |
| 23 | 0 | 30 061 | 59.7 | 43.3 | 66 | 50 | W. | ......... |
| 24 | 0 | 30.155 | 52.0 | 24.3 | 60 | 47 | N. W. | ......... |
| 25 | 0 | 30.238 | 52.5 | 30.0 | 61 | 40 | N. E. | ......... |
| 26 | 0 | 30.076 | 60.7 | 50.6 | 70 | 44 | S. E. | ......... |
| 27 | 08 | 29 703 | 64.5 | 67.7 | 70 | 58 | S. W. | ......... |
| 28 | 0 | 29.770 | 53.0 | 33.3 | 63 | 47 | W. | ......... |
| 29 | 0 | 30.056 | 50.2 | 42.3 | 58 | 40 | N. W. | ......... |
| 30 | 0 | 30.126 | 51.7 | 33.3 | 60 | 40 | N. W. | ......... |
| 31 | 0 | 30 077 | 59.0 | 29.7 | 69 | 41 | N. E. | ......... |
| Sums | 11 87 | 931.367 | 174.09 | 194.30 | 19.68 | 14.75 | ......... | ......... |

Highest Barometer, 29.623, on the 29th.
Lowest Barometer, 29.623, on the 27th.
Monthly range of Barometer, 0.745.
Highest Temp. 78°, on 7th. Lowest Temp. 35°, on 17th.
Monthly range of Temperature, 43°.
Greatest daily range of Temperature, 26°, on the 26th.
Least daily range of Temperature, 5°, on the 13th.
Mean of Maximum Temperatures, 63°.5
Mean of Minimum Temperatures, 47°.6
Mean daily range of Temperature, 15°9. Total rainfall, 11.87 inches.
Prevailing wind, N. W. Total movement of wind, 82.77 miles.
Maximum velocity of wind and direction, 37 miles per hour, W., 28th.
Number of cloudy days on which no rain fell, 11.
Total number of days on which rain fell, 15.

H. HALL, Corp'l. Signal Service U. S. A.

Atlanta, Ga., March 1, 1880.

